# Correlation of hippocampal atrophy with hyperhomocysteinemia in hemodialysis patients: An exploratory pilot study

**DOI:** 10.1371/journal.pone.0175102

**Published:** 2017-04-10

**Authors:** Kyoko Maesato, Takayasu Ohtake, Yasuhiro Mochida, Kunihiro Ishioka, Machiko Oka, Hidekazu Moriya, Sumi Hidaka, Shuzo Kobayashi

**Affiliations:** Department of Kidney Disease and Transplant Center, Shonan Kamakura General Hospital Kamakura, Kanagawa, Japan; Fraunhofer Research Institution of Marine Biotechnology, GERMANY

## Abstract

**Background:**

Cognitive impairment is one of the important critical issues in hemodialysis (HD) patients. However, the associating factors of brain atrophy in HD patients have not been fully elucidated.

**Purpose and methods:**

Brain magnetic resonance imaging (MRI) was performed in 34 of total 72 HD outpatients in our dialysis center. These MRI images were analyzed by an application software; Voxel-based Specific Regional Analysis System for Alzheimer’s Disease (VSRAD). VSRAD quantitatively calculates the extent of brain atrophy (percent of volume reduction) comparing with a MRI imaging database of 80 age-matched healthy controls. The extent of both hippocampal and whole-brain atrophy was evaluated with possible contributing factors.

**Results:**

In all patients, the mean extent of hippocampal atrophy was 27.3%, and the mean extent of whole-brain atrophy was 11.2%. The extent of hippocampal atrophy was significantly correlated with low body mass index (BMI), total serum homocysteine (tHcy) levels, and brachial-ankle pulse wave velocity (baPWV). The extent of whole-brain atrophy showed significant correlations with age, hypoalbuminemia, and baPWV. Based on the multiple regression analysis, tHcy was an independent determinant of hippocampal atrophy (β = 0.460, R^2^ = 0.189, P<0.01); while age was an independent determinant of whole-brain atrophy (β = 0.594, R^2^ = 0.333, P<0.01).

**Conclusions:**

In this exploratory pilot study, hippocampal atrophy was significantly correlated with hyperhomocysteinemia in HD patients.

## Introduction

Chronic kidney disease (CKD) is classified as one of the major risk factors for the progression of atherosclerotic disease [1]. Furthermore, CKD and atherosclerosis are forming vicious cycle with interacting each together. Higher serum levels in homocysteine (total homocysteine; tHcy) are also known to be as an independent risk factor for atherosclerotic disease [2]. In hemodialysis (HD) patients, serum tHcy levels are known to be highly elevated frequently [3].

A cognitive impairment is prevalent among dialysis patients [4]. According to a report of the Japanese Society for Dialysis Therapy, 9.9% of all dialysis patients had cognitive impairment [5]. The underlying pathogenesis of cognitive impairment could be numerous. However, at early phase of cognitive impairment, the ischemic change of capillary in cerebral parenchyma has been recognized. We have already reported a relationship between traditional atherosclerotic disease and cognitive impairment [6]. Brain ischemia due to atherosclerosis may simultaneously accelerate the progression of cognitive impairment and brain atrophy. It has been already known that cerebral atrophy is more severe in HD patients than in healthy volunteers [7]. However, the associating factors for advanced brain atrophy in HD patients have not been clarified.

In the present study, we investigated hippocampal atrophy level by magnetic resonance imaging (MRI). We also investigated factors that related to atrophy of both whole-brain and hippocampus in HD patients.

## Materials and methods

Tokushukai group ethical committee in the Mirai Iryo Research Center Inc. permits this study. Permission number of this study; TGE00396-061.

We got the informed consent from participants by both written and verbal methods. When we performed MRI, we got written informed consent after having explained significance of the inspection. Afterwards, before we used MRI imaging data in this study, we announced using poster presentation about the study in the dialysis center and gathered opt outs. This process was approved by ethical committee in our medical group. As a result, there were no participants who displayed opt out. Verbal consent was explained as follows; demented patients have been rapidly increasing along with aging of the population in Japan. Supporting diagnosis such as the MRI imaging is generally conducted to judge the risk of the dementia onset. Similarly in the dialysis field, there have been social problems about the dialysis introduction in case of high age patients, and the long-term dialysis patient with aging. We think it important to measure brain atrophy level of patients because of both maintaining dialysis treatment safely and preventing the progression of dementia in dialysis patients. We keep the written consent in a personal medical record. Our ethics committees/IRBs approved as Inclusion agreement.

### Patients

**Fig 1:**
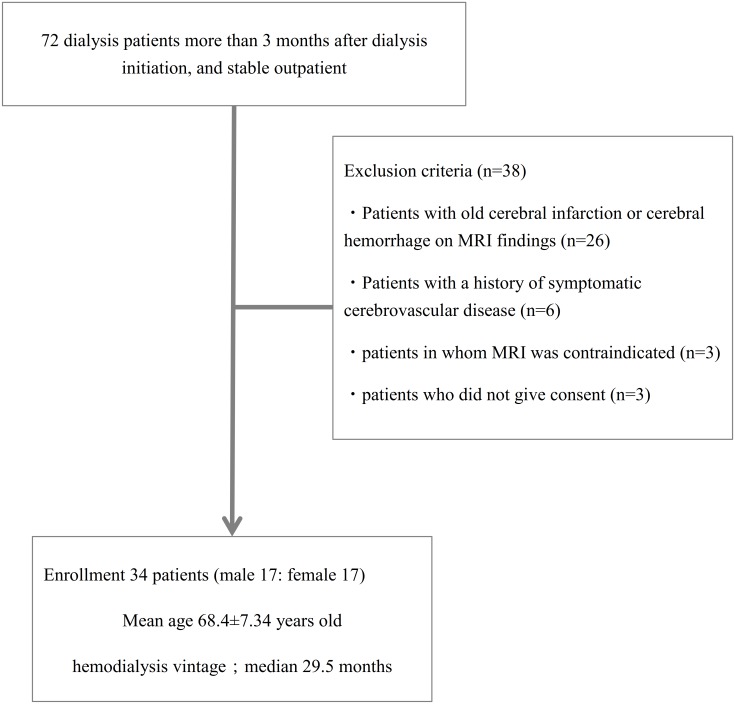
Patient enrollment flow in this study. 34 patients were enrolled in this study from 76 maintenance HD patients in our dialysis center.

This study was conducted in single HD center where a total of 72 stable outpatients received maintenance HD. We selected qualified patients who met all three following inclusion criteria, 1) prevalent HD patient with more than 3 month dialysis history, 2) patients without any hospitalization in preceding 3 months, and 3) patients received regular 4 hour HD three times a week. Between January 2010 and December 2010, 34 patients (17 males and 17 females, mean age: 68.4 years) were enrolled in this study after excluding the following cases; 1) patients in whom MRI was contraindicated, 2) patients with a history of symptomatic cerebrovascular disease, 3) patients with old cerebral infarction or cerebral hemorrhage on MRI, and 4) patients who did not give consent (Fig 1).

All patients underwent brain MRI, echocardiography for measurement of left ventricular mass index (LVMI), and brachial-ankle pulse wave velocity (baPWV) as a surrogate of arterial stiffness on non-dialysis day. In addition, measurements of blood pressure and pulse rate as well as collection of blood samples were done at the beginning of the first dialysis session of the week. Information concerning past history, and complications/pre-existing diseases (including hypertension (HTN), diabetes mellitus (DM), ischemic heart disease (IHD), and peripheral arterial disease (PAD); settled these diseases as ‘concurrent diseases’ in the following sentences) were collected from questionnaires and clinical records.

Concurrent diseases were defined as follows.

- HTN: pre-dialysis blood pressure (at the beginning of the week) over 140/90 mmHg or use of antihypertensive medications.
- DM: previous diagnosis or use of anti-diabetic drug.
- IHD: a history/treatment for myocardial infarction or angina pectoris.
- PAD: a history of revascularization therapy for lower limbs’ arteries or an ABI value below 0.9.

### VSRAD

Analysis of brain MRI images was performed to determine the extent of hippocampal (HP) atrophy and the extent of whole-brain (WB) atrophy using Voxel-based Specific Regional Analysis System for Alzheimer’s Disease (VSRAD plus^®^, Eisai, Japan) software. This software requires a three-dimensional volumetric acquisition of a T1-weighted gradient echo sequence of thin sagittal sections. VSRAD automatically analyzes MRI data as a series of segmentation, anatomical standardization and smoothing using SPM2 (Statistical Parametric Mapping 2002), and Z-score analysis. Specifically, after taking MRI imaging, VSRAD software automatically extracts the gray matter, and standardizes the brain shape of each subject. The software divides whole brain imaging into the voxel of the 2mm cube unit by the Voxel-based morphometry method, and analyzed the capacity of each voxel. The values of voxel, that mean gray matter bulk density with brightness, are calculated in each voxel level. VSRAD compared subject’s result with the normal control image, and calculated the percentage of HP atrophy (%HP extent) and WB atrophy (%WB extent). VSRAD has a database of 80 healthy Japanese male and female volunteers aged between 54 to 86 years (mean 70.1, S.D. 7.7). Healthy volunteers showed normal levels in memory and the cognitive function test by both Wechsler Memory Scale-Revised and Wechsler Adult Intelligence Scale-Revised. Their mini-mental state examination (MMSE) score ranged from 26 to 30; mean 28.7, S.D. 1.5.Then Z-score; ([(normal control average of voxel level—patient’s voxel level)/ (normal control standard deviation)]) are calculated in each voxel and judged as atrophy if the deletional expanse of the voxel more than Z-score 2. The severity of volume reduction was expressed with percent volume loss (e.g.; when there is no atrophy, the value is 0%). Calculating formula was as follows; (number of the voxels judged more than Z-score 2) / (number of all voxel in the VOI (volume of interest, in this case, both two region: hippocampal, parahippocampal gyrus area and whole brain)) ×100(%). The validity of VSRAD software has already been reported elsewhere [8, 9].

We used MRI device with strength of magnetic field 1.5 tesra (SIGNA EXCITE^®^, version 11.1, GE Healthcare, Waukesha, Wisconsin, USA).

### Statistical analysis

Data was expressed as mean±standard deviation (SD). Skewed data including tHcy, high sensitive C-reactive protein (hsCRP), and %HP extent or %WB extent were converted to logarithmic values before the analysis. Then, the correlation between log %HP extent and log %WB extent was analyzed with several clinical and physiological parameters. When a single correlation was significant, the factor was used to multiple regression analysis. We also tried to define the associating factors of hippocampal atrophy using 3 different models, model 1, model 2, and model 3. Possible variables of hippocampal atrophy included age, HD vintage, and DM in model 1, model 1 plus nutritional and inflammatory variables including serum albumin, BMI, and hsCRP in model 2, and model 2 plus atherosclerotic surrogates including baPWV and tHcy in model 3. In all analyses, P<0.05 was considered to be significant.

## Results

### Patients

Clinical characteristics of the patients, concurrent diseases and their prescription drugs are shown in Table 1. The median duration of dialysis was 29.5 months. Concurrent diseases were as following; DM (n = 14 (41%)), HTN (n = 32(94%)), IHD (n = 6(17%)), and PAD (n = 8(23%)). The mean pre-dialysis blood pressure at the beginning of the week were 152/78 mmHg. The results of blood tests for HD status, nutritional status, and inflammatory parameters were on relatively stable condition (Table 1).

**Table 1:** Baseline characteristics of the patients.

	Mean±SD or median (IQR)	Reference ranges
**Age (years old)**	68.4±7.34	
**Gender**	Male: Female = 17:17	
**Cause of renal failure, n (%)**	Diabetic nephropathy 13 (38.2) Chronic glomerulonephritis 9 (26.5) Nephrosclerosis 4 (11.8) Chronic pyelonephritis 2 (5.9) Unknown 3 (8.8)	
**Hemodialysis duration (month)**	29.5 (17–65.5)	
**BMI (kg/m**^**2**^**)**	21.8±3.3	
**Diabetes Mellitus, n (%)**	14 (41.2)	
**Hypertension, n (%)**	32 (94.1)	
**Ischemic heart disease, n (%)**	6 (17.6)	
**Peripheral arterial disease, n (%)**	8 (23.5)	
**Calcium Channel Blocker use, n (%)**	16 (47.1)	
**RAS inhibitor (ACE-I, ARB) use, n (%)**	20 (58.8)	
**Active form vitamin D use, n (%)**	26 (76.5)	
**Pre dialysis systolic BP(mmHg)**	152±23	
**Pre dialysis diastolic BP(mmHg)**	77±11	
**Pulse pressure(mmHg)**	74.4±17.2	
**Total Protein(g/dl)**	6.40±0.58	6.4–8.3
**Albumin(g/dl)**	3.80±0.34	3.8–5.2
**Corrected Ca (mg/dl)**	8.98±0.48	8.3–10.4
**IP (mg/dl)**	5.71±1.35	2.7–4.5
**Ca×IP (mg**^**2**^**/dl**^**2**^**)**	51.3±12.4	
**Intact PTH (pg/ml)**	177.1±122.4	8.7–79.5
**Hemoglobin(g/dl)**	11.0±1.2	11.0–15.6
**Platelet (×10**^**4**^**/μl)**	17.6±5.5	12.4–30.5
**hs-CRP (mg/dl)**	0.11(0.04–0.38)	<0.5
**Log hs-CRP**	-0.92±0.6	
**Fibrinogen (mg/dl)**	366.2±68.8	150–400
**β**_**2**_**microgloblin (mg/l)**	25.3±6.5	0.9–1.9
**tHcy (μmol/l)**	27.6(20.5–52.7)	5.1–11.7
**Log tHcy**	1.55±0.32	

Abbreviations are; BMI, body mass index; RAS, renin-angiotensin system; ACE-I, angiotensin-converting enzyme inhibitor; ARB, angiotensin type2 receptor blocker; BP, blood pressure; Ca, calcium; IP, inorganic phosphate; PTH, parathyroid hormone; hs-CRP, hypersensitive C-reactive protein; tHcy, total homocysteine; Log tHcy, logarithmic total homocysteine.

### %HP extent, %WB extent and related parameters

**Fig 2:**
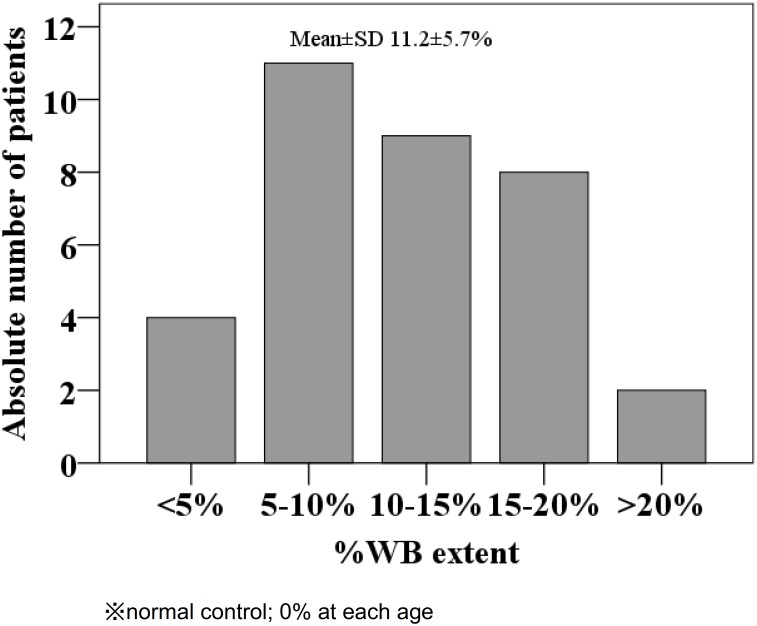
Distribution of %WB extent. %WB extent showed normal distribution.

**Fig 3:**
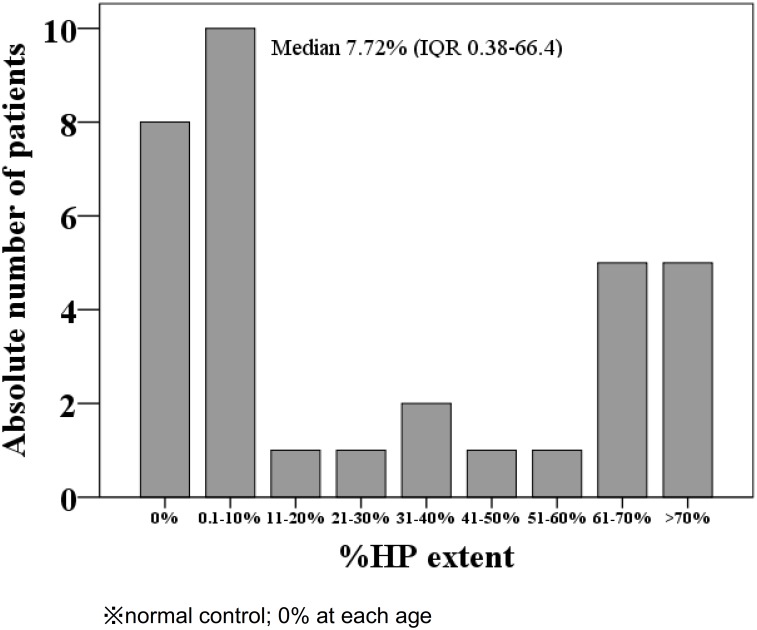
Distribution of % HP extent. %HP extent showed U-shaped distribution.

Mean %WB extent showed 11.2% (median 11.3%) with a normal distribution (Fig 2). However, %HP extent did not show normal distribution, but U-shaped distribution in which 8 patients were without hippocampal atrophy (Fig 3). The mean±SD baPWV was 1729±290 cm/second with serum tHcy levels of 48.9±48.1 nmol/mL (median 27.6 nmol/mL) which was more than twice the upper limit of normal distribution (19 nmol/mL) (Table 2).

**Table 2:** Results of %HP extent, %WB extent and surrogates of atherosclerosis.

	Mean±SD or median(IQR)	Reference ranges
**%HP extent (%)**	7.72 (0.38–66.4)	0
**Log %HP extent**	0.45±1.51	
**%WB extent (%)**	11.2±5.7	0
**Log %WB extent**	0.99±0.26	
**LVMI (g/m**^**2**^**)**	149.3±38.5	Male<125 Female<110
**ABI**	1.16±0.17	0.9–1.3
**baPWV (cm/s)**	1729.8±290.3	
**TBI**	0.74±0.18	>0.6

HD patient showed median %HP extent 7.72%, and median %WB extent 11.2%. Also, they showed high levels of serum tHcy and LVMI.

Abbreviations are: HP, hippocampus; WB, whole-brain; LVMI, left ventricular mass index; ABI, ankle brachial index; baPWV, brachial-ankle pulse wave velocity; TBI, tibial brachial index

**Fig 4:**
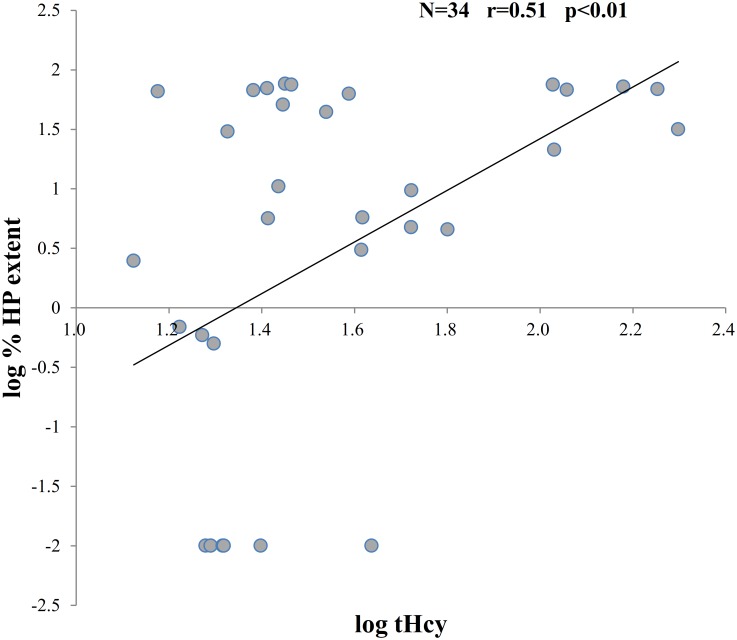
Correlation between log %HP extent and log tHcy. Log %HP extent was strongly associated with log tHcy level linearly.

By single correlation analysis, % HP extent showed significant correlations with low body mass index (BMI), logarithmic tHcy, and baPWV (Table 3). With regard to the %WB extent, it showed significant correlations with age, hypoalbuminemia, and baPWV (Table 3). In multiple regression analysis, logarithmic tHcy was independently associated with logarithmic %HP extent (β = 0.460, R^2^ = 0.189, P<0.01) (Fig 4), while age (β = 0.594, R^2^ = 0.333, P<0.01) was independently associated with logarithmic %WB extent (β = 0.594, R^2^ = 0.333, P<0.01) (Table 3).

**Table 3:** Correlation of %HP extent and %WB extent with several factors.

**%HP extent**				
	**Single correlation analysis**		**Multiple linear regression analysis**	
	***r***	***P***	**β**	**P**
**Log tHcy**	0.51	**<0.01**	0.46	**<0.01**
**BMI**	-0.38	**0.03**	-0.28	0.08
**baPWV**	0.43	**0.01**	0.23	0.16
**%WB extent**				
	**Single correlation analysis**		**Multiple linear regression analysis**	
	***r***	***P***	**β**	**P**
**Age**	0.65	**<0.01**	0.60	**<0.01**
**sAlb**	-0.48	**<0.01**	-0.25	0.11
**baPWV**	0.61	**<0.01**	0.14	0.47

%HP extent was related to high log tHcy level. On the other hand, %WB extent was related to high age, significantly.

Abbreviations are; %HP, percentage of hippocampal atrophy; %WB, percentage of whole-brain atrophy; tHcy, total homocysteine; BMI, body mass index; baPWV, brachial-ankle pulse wave velocity; sAlb, serum albumin.

### Comparison with or without hippocampal atrophy

Eight patients without hippocampal atrophy showed significantly lower serum tHcy concentration compared with those in 26 patients with hippocampal atrophy (tHcy; 23.6±8.2 vs. 56.7±52.6, *P*<0.01); however, serum inorganic phosphate (IP) (6.7±1.6 vs. 5.4±1.1 mg/dl, *P* = 0.01), BMI (23.9±4.1 vs. 21.1±2.7 kg/m^2^, *P* = 0.03), and calcium (Ca) x IP (59.3±14.4 vs. 48.8±10.9 mg^2^/dl^2^, *P* = 0.04) were significantly higher in patients without hippocampal atrophy compared with those in patients who had hippocampal atrophy (Table 4).

**Table 4:** Comparison with or without hippocampal atrophy.

	atrophy (-) (n = 8)	atrophy (+) (n = 26)	*P*
**Age (years old)**	65.0±9.9	69.4±6.2	0.264
**Gender (Male: Female)**	3:5	14:12	0.419
**Hemodialysis duration (month)**	57.6±104	58.6±60.9	0.974
**BMI (kg/m**^**2**^**)**	23.9±4.1	21.1±2.7	**0.034**
**Diabetes Mellitus, n (%)**	4 (50)	10 (38.5)	0.562
**Hypertension, n (%)**	8 (100)	24 (92)	0.419
**Ischemic heart disease, n (%)**	1 (13)	5 (19.2)	0.662
**Peripheral arterial disease, n (%)**	3 (38)	5 (19)	0.287
**Calcium Channel Blocker use, n (%)**	2 (25)	14 (54)	0.153
**RAS inhibitor (ACE-I, ARB) use, n (%)**	4 (50)	16 (61.5)	0.562
**Active form vitamin D use, n (%)**	5 (62.5)	21 (80.8)	0.287
**Pre dialysis systolic BP(mmHg)**	159±28	150±22	0.360
**Pre dialysis diastolic BP(mmHg)**	83±12	76±11	0.179
**Pulse pressure(mmHg)**	76.3±23.6	73.8±15.3	0.731
**Total Protein(g/dl)**	6.4±0.36	6.4±0.64	0.944
**Albumin(g/dl)**	3.9±0.26	3.8±0.36	0.300
**Corrected Ca (mg/dl)**	8.8±0.35	9.0±0.51	0.345
**IP (mg/dl)**	6.7±1.6	5.4±1.1	**0.013**
**Ca×IP (mg**^**2**^**/dl**^**2**^**)**	59.3±14.4	48.8±11.0	**0.035**
**Intact PTH (pg/ml)**	189.4±174	173.3±106	0.751
**Hemoglobin(g/dl)**	10.9±1.6	11.1±1.1	0.688
**Platelet (×10**^**4**^**/μl)**	18.8±8.8	17.2±5.9	0.481
**Log hs-CRP**	-0.9±0.6	-0.9±0.6	0.996
**Fibrinogen (mg/dl)**	349.6±64	371.2±70.6	0.448
**β**_**2**_**microgloblin (mg/l)**	23.3±2.0	25.9±7.2	0.318
**Log %WB extent**	0.91±0.19	1.01±0.27	0.318
**tHcy (B exten**	23.6±8.2	56.7±52.6	**0.004**
**Log tHcy**	1.36±0.12	1.61±0.34	**0.003**
**LVMI (g/m**^**2**^**)**	142±29	152±41	0.549
**ABI**	1.2±0.16	1.1±0.17	0.467
**baPWV (cm/s)**	1648±290	1757±265	0.327
**TBI**	0.79±0.17	0.73±0.18	0.429

Patients without hippocampal atrophy were related to high BMI, high serum IP level, high Ca×IP level, low tHcy level, and low log tHcy, significantly.

Abbreviations are; BMI, body mass index; RAS, renin-angiotensin system; ACE-I, angiotensin-converting enzyme inhibitor; ARB, angiotensin type2 receptor blocker; BP, blood pressure; Ca, calcium; IP, inorganic phosphate; PTH, parathyroid hormone; hs-CRP, hypersensitive C-reactive protein; WB, whole-brain; tHcy, total homocysteine; LVMI, left ventricular mass index; ABI, ankle brachial index; baPWV, brachial-ankle pulse wave velocity; TBI, tibial brachial index

### Multiple regression analysis of %HP extent and concurrent diseases, associated factors

We made three models to adjust the concurrent important diseases and the above-mentioned factor concerned. Multiple regression analysis is shown in Table 5.

**Table 5:** Multiple regression analysis about %HP extent.

	Model 1		Model 2		Model 3	
	β	*P*	β	*P*	β	*P*
**Age**	0.27	0.134	0.22	0.301	0.23	0.359
**HD vintage**	-0.21	0.257	-0.18	0.329	-0.12	0.489
**DM**	-0.1	0.592	-0.10	0.605	-0.04	0.836
**Alb**			-0.16	0.445	-0.26	0.203
**log hsCRP**			-0.26	0.223	-0.22	0.264
**BMI**			-0.23	0.274	-0.11	0.562
**baPWV**					0.001	0.998
**log tHcy**					0.44	**0.023**

%HP extent was strongly related to the logarithmic tHcy.

Model 1: Adjusted with age, HD vintage, and DM

Model 2: Adjusted with model 1 plus Alb, log hsCRP, BMI

Model 3: Adjusted with model 2 plus baPWV, log tHcy

Abbreviations are; %HP, percentage of hippocampal atrophy; HD, hemodialysis; DM, diabetes mellitus; Alb, albumin; hsCRP, hypersensitive C-reactive protein; BMI, body mass index; baPWV, brachial-ankle pulse wave velocity; tHcy, total homocysteine.

Model 1 contained basic factors such as age, HD vintage, and DM. In model 2, inflammation and nutritional parameters were added to model 1; serum albumin concentration, logarithmic hsCRP, and BMI. In model 3, atherosclerotic parameters were added to model 2; baPWV and logarithmic tHcy concentration. Even in the case of multiple linear regression analysis that includes concurrent diseases, only logarithmic tHcy concentration is related to the %HP extent (β = 0.44, *P*<0.05) (Table 5).

## Discussion

Our cross-sectional exploratory pilot study showed that the %HP extent was correlated with BMI, tHcy, and baPWV in a single correlation analysis, of which tHcy was an independent determinant of %HP extent. The %WB extent was correlated with age, hypoalbuminemia, and baPWV as well. Among these parameters, age was an independent determinant of %WB extent.

There have been already many reports concerning the relationship between cerebral atrophy and aging in non-dialysis patients [7]. The present study also showed that %WB extent was highly associated with age also in HD patients. We identified 8 HD patients without hippocampal atrophy. Hippocampal atrophy was reported to be more prominent in hypertensive patients [10]. However, there was no significant difference in both systolic and diastolic blood pressure levels between our HD patients with or without hippocampal atrophy (Table 4).

Moreover, multiple regression analysis showed significantly positive association between %HP extent and serum tHcy levels. Although limited, a few reports have been published concerning the relationship between higher serum level of tHcy with cognitive impairment [11] and hippocampal atrophy [12] in non-dialysis patients. Serum tHcy usually shows abnormally elevated levels in HD patients [13]. It is clearly reported that serum tHcy significantly and inversely correlates with renal function [14].

Hyperhomocysteinemia is known to adversely affect endothelial cells, and neurons. These mechanisms are: first, homocysteine induces DNA damage/apoptosis due to oxidative stress mediated via an increase of intracellular H_2_P_2_/caspase 3 [15]; second, vascular endothelial cell damage by increasing tissue factors/ICAM-1 [16]; third, thrombus formation via both platelet activation and decreased thrombomodulin/protein C activation [17]; and fourth, direct neurotoxicity via promotion of pro-apoptotic signaling and increased sensitivity to extracellular toxins [18]. Hyperhomocysteinemia is thought to affect the hippocampus more strongly than any other part of the brain. It has been recently reported that hyperhomocysteinemia affects the cerebral smaller vessels more than major vessels [19]. Blood of hippocampal region is supplied via the anterior choroidal artery, which arises from distal to the bifurcation of the posterior communicating artery, and is classified as a perforator branch. Although the diameter of anterior choroidal artery is relatively large (being 0.7–2.0 mm), thrombosis and thrombotic infarction are more likely to occur in this vessel because of its long course through the subarachnoid space.

We recently studied the relationship between the regional cerebral blood flow (rCBF) by quantitative single-photon emission-computed tomography (SPECT) and various parameters including cognitive function test (MMSE) in patients on HD [6] and also peritoneal dialysis (PD) [20]. More than half of HD patients (63%) showed normal range of MMSE scores more than 28 (full score 30). However, rCBF of all HD patients’ studies was significantly lowered compared with rCBF of normal control patients [6]. Moreover, we provided ischemic changes of parahippocampal lesion in these very sensitive and detailed brain perfusion studies [6, 20].

The limitations of this study were 1) a single center cross-sectional observational study with a small number of Japanese patients, 2) lack of nutritional assessment, and 3) lack of cognitive function test. The main limitation of this study is that cognitive impairment was not assessed and we could not know anything of its presence or severity in the studied group. Measuring of brain atrophy without cognitive assessment is not enough, but preventing strategies may be or should be implemented in every patient regardless of the diagnosis, of course especially in a group with higher risk of developing dementia like patients with kidney failure treated with hemodialysis. Our present exploratory pilot study indicated the close relationship between hippocampal atrophy and hyperhomocysteinemia in HD patients. However, small sample size made it difficult to conclude definitely. In a future research, it will be needed to assess cognitive function and nutritional status along with brain atrophy in more large scale.

## Conclusions

In conclusion, tHcy was an independent determinant of hippocampal atrophy while age was an independent determinant of whole-brain atrophy in this exploratory pilot study. Although we could not provide the causative relationship, our study showed association between hyperhomocysteinemia and hippocampal atrophy. Because hippocampal and whole-brain atrophy is thought to be associated with cognitive dysfunction, influence of serum tHcy levels on longitudinal changes of hippocampal atrophy and cognitive function should be clarified in the future.

## References

[1] Balla S, Nusair MB, Aloert MA. Risk factors for atherosclerosis in patients with chronic kidney disease: recognition and management. Cur Opin Pharmacol. 2013; 13(2): 192–199. 10.1016/j.coph.2012.12.001 23291030

[2] Kampoli AM, Tousoulis D, Papageorgiou N, Pallatza Z, Vogiatzi G, Briasoulis A, et.al Clinical utility of biomarkers in premature atherosclerosis. Curr Med Chem 2012; 19(16): 2521–2533. 22489712 10.2174/092986712800493039

[3] Righetti M. Cardio-renal-anemia syndrome: a link between erythropoietin, dimethylarginine and homocyteine. Curr Med Chem 2012; 19(21): 3502–3507. 22680636 10.2174/092986712801323261

[4] Post JB, Morin KG, Sano M, Jagede AB, Langhoff E, Spungen AM. Increased presence of cognitive impairment in hemodialysis patients in the absence of neurological events. Am J Nephrol 2012; 35: 120–126. 10.1159/000334871 22212437 PMC3711004

[5] Nakai S, Iseki K, Itami N, Ogata S, Kazama JJ, Kimata N, et al Overview of regular dialysis treatment in Japan (as of Dec. 31, 2009). Ther Apher Dial 2012; 16(1): 11–53. 10.1111/j.1744-9987.2011.01050.x 22248195

[6] Kobayashi S, Mochida Y, Ishioka K, Oka M, Maesato K, Moriya H, et al The effects of blood pressure and the renin-angiotensin- aldosterone system on regional cerebral blood flow and cognitive impairment in dialysis patients. Hypertens Res 2014; 37(7): 636–641. 10.1038/hr.2014.57 24694648

[7] Kamata T, Hishida A, Takita T, Sawada K, Ikegaya N, Maruyama Y, et al Morphologic abnormalities in the brain of chronically hemodialyzed patients without cerebrovascular disease. Am J Nephrol 2000; 20(1): 27–31. doi: 13551 10644864 10.1159/000013551

[8] Hirata Y, Matsuda H, Nemoto K, Ohnishi T, Yamashita F, Asada T, et al Voxel-based morphometry to discriminate early Alzheimer’s disease from controls. Neurosci Lett. 2005; 382(3): 269–274. 10.1016/j.neulet.2005.03.038 15925102

[9] Matsuda H, Mizumura S, Nemoto K, Yamashita F, Imabayashi E, Sato N, et al Automatic voxel-based morphometry of structural MRI by SPM8 plus diffeomorphic anatomic registration through exponentiated lie algebra improves the diagnosis of probable Alzheimer disease. Am H Neuroradiol. 2012; 33: 1109–1114. 10.3174/ajnr.A2935 PMC8013247 22300935

[10] Beauchet O, Celle S, Roche F, Bartha R, Montero-Odasso M, Allali G, et al Blood pressure levels and brain volume reduction: a systematic review and meta-analysis. J Hypertens. 2013; 31(8): 1502–1516. 10.1097/HJH.0b013e32836184b5 23811995

[11] Cacciapuoti F. Lowering homocysteine levels with folic acid and B-vitamins do not reduce early atherosclerosis, but could interfere with cognitive decline and Alzheimer’s disease. J Thromb Thrombolysis 2013; 36(3): 258–262. 10.1007/s11239-012-0856-x 23224755

[12] Shimomura T, Anan F, Masaki T, Umeno Y, Eshima N, Saikawa T, et al Homocysteine levels are associated with hippocampus volume in type 2 diabetic patients. Eur J Clin Invest 2011; 41(7): 751–758. 10.1111/j.1365-2362.2010.02464.x 21250986

[13] Jardine MJ, Kang A, Zoungas S, Navaneethan SD, Ninomiya T, Nigwekar SU, et al The effect of folic acid based homocysteine lowering on cardiovascular events in people with kidney disease: systematic review and meta-analysis. BMJ 2012; 344: e3533 10.1136/bmj.e3533 22695899 PMC3374481

[14] Arnadottir M, Hultberg B, Nilsson-Ehle P, Thysell H. The effect of reduced glomerular filtration rate on plasma total homocysteine concentration. Scand J Clin Lab Invest 1996; 56(1): 41–46. 10.3109/00365519609088586 8850171

[15] Huang RF, Huang SM, Lin BS, Wei JS, Liu TZ. Homocysteine thiolactone induces apoptotic DNA damage mediated by increased intracellular hydrogen peroxide and caspase 3 activation in HL-60 cells. Life Sci 2001; 68(25): 2799–2811 11432446 10.1016/s0024-3205(01)01066-9

[16] Hassan A, Hunt BJ, O’Sullivan M, Bell R, D’Souza R, Jeffery S, et al Homocysteine is a risk factor for cerebral small vessel disease, acting via endothelial dysfunction. Brain 2004; 127: 212–219. 10.1093/brain/awh023 14607791

[17] Rodgers GM, Conn MT. Homocysteine, an atherogenic stimulus, reduces protein C activation by arterial and venous endothelial cells. Blood 1990; 75(4): 895–901. 2154269

[18] Kruman II, Culmsee C, Chan SL, Kruman Y, Guo Z, Penix L, et al Homocysteine elicits a DNA damage response in neurons that promotes apoptosis and hypersensitivity to excitotoxicity. J Neurosci 2000; 20(18): 6920–6926. 10995836 10.1523/JNEUROSCI.20-18-06920.2000 PMC6772815

[19] Kloppenborg RP, Geerlings MI, Mali WPTM, Vermeulen M, Graaf Y, Nederkoorn PJ, et al Homocysteine and progression of generalized small-vessel disease. The SMART-MR study. Neurology 2014; 82: 777–783. 10.1212/WNL.0000000000000168 24477110

[20] Isshiki R, Kobayashi S, Iwagami M, Tsutumi D, Mochida Y, Ishioka K, et al Cerebral blood flow in patients with peritoneal dialysis by an easy Z-score imaging system for brain perfusion single-photon emission tomography. Ther Apher Dial. 2014; 18(3): 291–296. 10.1111/1744-9987.12107 24965295

